# Existing Barriers Faced by and Future Design Recommendations for Direct-to-Consumer Health Care Artificial Intelligence Apps: Scoping Review

**DOI:** 10.2196/50342

**Published:** 2023-12-18

**Authors:** Xin He, Xi Zheng, Huiyuan Ding

**Affiliations:** 1 School of Mechanical Science and Engineering Huazhong University of Science and Technology Wuhan China

**Keywords:** artificial intelligence, medical, health care, consumer, consumers, app, apps, application, applications, DTC, direct to consumer, barrier, barriers, implementation, design, scoping, review methods, review methodology

## Abstract

**Background:**

Direct-to-consumer (DTC) health care artificial intelligence (AI) apps hold the potential to bridge the spatial and temporal disparities in health care resources, but they also come with individual and societal risks due to AI errors. Furthermore, the manner in which consumers interact directly with health care AI is reshaping traditional physician-patient relationships. However, the academic community lacks a systematic comprehension of the research overview for such apps.

**Objective:**

This paper systematically delineated and analyzed the characteristics of included studies, identified existing barriers and design recommendations for DTC health care AI apps mentioned in the literature and also provided a reference for future design and development.

**Methods:**

This scoping review followed the Preferred Reporting Items for Systematic Reviews and Meta-Analyses Extension for Scoping Reviews guidelines and was conducted according to Arksey and O’Malley’s 5-stage framework. Peer-reviewed papers on DTC health care AI apps published until March 27, 2023, in Web of Science, Scopus, the ACM Digital Library, IEEE Xplore, PubMed, and Google Scholar were included. The papers were analyzed using Braun and Clarke’s reflective thematic analysis approach.

**Results:**

Of the 2898 papers retrieved, 32 (1.1%) covering this emerging field were included. The included papers were recently published (2018-2023), and most (23/32, 72%) were from developed countries. The medical field was mostly general practice (8/32, 25%). In terms of users and functionalities, some apps were designed solely for single-consumer groups (24/32, 75%), offering disease diagnosis (14/32, 44%), health self-management (8/32, 25%), and health care information inquiry (4/32, 13%). Other apps connected to physicians (5/32, 16%), family members (1/32, 3%), nursing staff (1/32, 3%), and health care departments (2/32, 6%), generally to alert these groups to abnormal conditions of consumer users. In addition, 8 barriers and 6 design recommendations related to DTC health care AI apps were identified. Some more subtle obstacles that are particularly worth noting and corresponding design recommendations in consumer-facing health care AI systems, including enhancing human-centered explainability, establishing calibrated trust and addressing overtrust, demonstrating empathy in AI, improving the specialization of consumer-grade products, and expanding the diversity of the test population, were further discussed.

**Conclusions:**

The booming DTC health care AI apps present both risks and opportunities, which highlights the need to explore their current status. This paper systematically summarized and sorted the characteristics of the included studies, identified existing barriers faced by, and made future design recommendations for such apps. To the best of our knowledge, this is the first study to systematically summarize and categorize academic research on these apps. Future studies conducting the design and development of such systems could refer to the results of this study, which is crucial to improve the health care services provided by DTC health care AI apps.

## Introduction

The scarcity and uneven distribution of health care resources, such as medical facilities and professionals, often impedes people’s access to timely and effective health care services and professional medical advice, which has been a significant health concern worldwide [1]. The World Health Organization (WHO) and other institutions have identified artificial intelligence (AI) as a technology that has the potential to fundamentally transform health care and help address these challenges, especially the reduction in health inequalities in low- and middle-income countries (LMICs) [2,3].

Among AI programs that provide health care functions, there is a significant surge in health care apps that are sold directly to consumers for personal use. Most of these apps are based on predictive or diagnostic functions, providing consumers with a purportedly inexpensive and accurate diagnosis of various conditions [4]. A well-known example is the Apple Watch for atrial fibrillation, which has been authorized as a class II (moderate-risk) device [5]. The increased emphasis on telemedicine and home health care in the era of the COVID-19 pandemic [6], as well as the current advancements in generative AI technologies, such as ChatGPT (where GPT stands for Generative Pretrained Transformer), further stimulate and drive the emergence of direct-to-consumer (DTC) health care AI apps. Large enterprises are racing to deploy research and development of DTC health care AI apps. For example, Dr Karen DeSalvo, Google’s chief health officer, argued at “Check Up 2023” that the future of health is consumer driven. As a company with advanced AI technologies, Google will drive AI-enabled insights, services, and care across a range of health care use cases, from search to symptom tracking and treatment [7].

However, on the one hand, existing DTC health care AI apps carry risks of errors at both the individual and the societal level. At the individual level, consumers may face the costs and consequences of overdiagnosis or underdiagnosis when using these apps. For example, Google announced an AI-powered dermatology assist app that, according to the company, can use deep learning to identify 288 skin, hair, and nail conditions based on user-submitted images [8]. However, the app has a significant limitation due to its lack of data diversity, which could lead to overdiagnosis or underdiagnosis in non-White patients [9]. At the societal level, DTC health care AI apps are designed for cost-effective, immediate, and repeated use, increasing the likelihood that their errors will spread rapidly and place a significant burden on the overall health care system [4].

On the other hand, the manner in which consumers interact directly with AI in DTC health care AI apps is transformative and alters the traditional physician-patient relationships. These apps can directly provide consumers with various functions, such as heart dysfunction identification [10,11], eye disease diagnosis [12], and emotion regulation and treatment [13], which were previously provided by human health care experts. However, in the process of consumers directly interacting with AI, failure to incorporate consumer behavior insights into AI technological development will undermine their experience with AI [14], thereby affecting their adoption of such apps [15].

In the context of a surge in DTC health care AI apps, academic research focusing on consumers in the health care AI field is relatively scarce, and there is limited understanding of consumer acceptance of AI in the health care domain [16]. Furthermore, most trials of clinical AI tools omit the evaluation of patients’ attitudes [17]. The majority of existing reviews either concentrate on health care AI systems for expert users, such as health care providers [18,19], or do not clearly differentiate the user categories for AI apps in health care [20,21]. There is a need for a deeper understanding of how consumers interact with DTC health care AI apps, beyond merely considering the system’s technical specifications [4]. Previous studies have reviewed AI apps that are patient oriented and have unique features, functionalities, or formats [22-24]. However, the overall landscape of DTC health care AI apps in academic research remains unclear. There is also a lack of studies that systematically summarize the potential barriers faced by these apps, as well as design recommendations for future research.

To the best of our knowledge, this is the first academic study to systematically summarize and sort out the profile of health care AI apps directly targeting consumers. The objectives of this research are twofold: first, to provide a comprehensive overview of existing studies related to DTC health care AI apps, exploring and mapping out their study characteristics, and, second, to summarize observed barriers and future design recommendations in the literature. Understanding these issues is crucial for the future research, design, development, and adoption of DTC health care AI apps.

## Methods

### Study Design

A scoping review was conducted in line with Arksey and O’Malley’s 5-stage framework [25]. Study results were reported according to the PRISMA-ScR (Preferred Reporting Items for Systematic Reviews and Meta-Analyses Extension for Scoping Reviews) checklist [26] (Multimedia Appendix 1).

### Stage 1: Identifying the Research Question

To address the aim of this study, 3 research questions were formulated:

- Research question 1: What characteristics of DTC health care AI apps have been identified in existing research?
- Research question 2: What barriers are faced by DTC health care AI apps in existing research?
- Research question 3: What design recommendations for DTC health care AI Apps have been put forward in existing research?

### Stage 2: Identifying Relevant Studies

Studies were searched from inception until March 27, 2023. We searched 5 databases (Web of Science, Scopus, the ACM Digital Library, IEEE Xplore, and PubMed) for 4 concept areas and their lexical variants and synonyms (Textbox 1): AI (technical basis), health care (application domain), consumer (user), and app (carrier). In addition, we retrieved gray literature from the top 10 pages of Google Scholar search results. Gray literature encompasses the literature produced by various levels of government, academia, business, and industry in both print and electronic formats, which is not controlled by commercial publishers [27]. Its forms include academic papers, dissertations, research and committee reports, government publications, conference papers, and ongoing research, among others.

**Concept areas and lexical variants and synonyms used to develop the search strategy.**

**Search concepts combined using “AND”**

- Artificial intelligence (AI)
- Health care
- Consumer
- App

**Search terms combined using “OR”**

- AI, artificial intelligence, ML, machine learning, DL, deep learning
- Health care, health, medical
- Consumer, consumers
- Application, applications, app, apps, system, systems, service, mHealth, eHealth

We also conducted snowball sampling on the reference lists of related papers included in the full-text review. The specific database search strings combined with Boolean operators are detailed in Multimedia Appendix 2.

### Stage 3: Study Selection

Inclusion criteria for this review were (1) peer-reviewed studies, (2) research papers, (3) papers published in English, (4) research topics focused on DTC health care AI apps or systems, and (5) either consumers as target users or multistakeholder users with consumers as main users. Exclusion criteria were (1) duplicate papers not identified by bibliography software, (2) nonresearch papers (eg, editorials, commentaries, perspectives, opinion papers, or reports), (3) papers not published in English, (4) inability to obtain the full text, and (5) app only intended to be used by professionals.

Inclusion and exclusion criteria (Table 1) were used to screen titles, abstracts, and full-text papers. When the 2 authors (XH and XZ) disagreed on the selection of studies, consensus was reached through discussion.

**Table 1:** Eligibility criteria.

Inclusion criteria	Exclusion criteria
Peer reviewed	Duplicate (not detected by bibliography software)
Research papers	Editorials, commentaries, perspectives, opinion papers, or reports
English language	Not presented in English language
Research topics related to DTC^a^ health care AI^b^ apps or systems	Full text not available
Consumers as target users or multistakeholder users with consumers as main users	App only intended to be used by professionals

^a^DTC: direct to consumer.

^b^AI: artificial intelligence.

### Stage 4: Charting the Data

Two authors (XH and XZ) extracted the following data for each paper: title, author, publication year, country, publication type, study objective, study design, medical field, app type, user, existing barriers, and design recommendations. We exclusively extracted data related to barriers and design recommendations from the results or discussions within the papers (eg, insights, such as opinions expressed by consumers after using the apps or recommendations proposed by researchers following app evaluations). Descriptions that were not validated through the empirical research section of the papers were not extracted (eg, viewpoints that appeared only in the Introduction or Background section).

### Stage 5: Collating, Summarizing, and Reporting Results

The extracted data related to RQ1 were mapped and summarized. A reflexive thematic analysis [28-30] was conducted on the data related to RQ2 and RQ3 to summarize existing barriers faced by and design recommendations for DTC health care AI apps through inductive coding. NVivo (QSR International) was used to facilitate data management and analysis. The analysis proceeded through 6 steps: familiarizing with the data set; coding; generating initial themes; developing and reviewing themes; refining, defining. and naming themes; and writing up. The coding and data analysis for this study were performed in parallel, and we addressed differences and reached consensus by discussing uncertainties.

## Results

### Search Results

**Figure 1:**
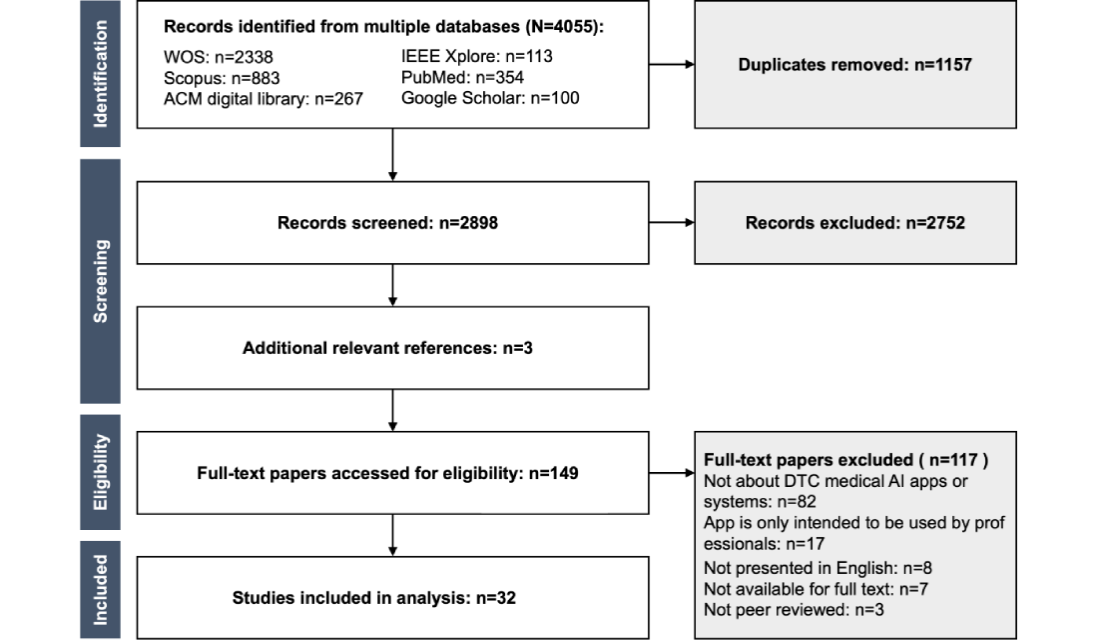
PRISMA-ScR flow diagram. We retrieved 4055 papers published until March 27, 2023, from 6 databases and ultimately included 32 (0.8%) papers after applying predetermined inclusion and exclusion criteria. AI: artificial intelligence; DTC: direct to consumer; PRISMA-ScR: Preferred Reporting Items for Systematic Reviews and Meta-Analyses Extension for Scoping Reviews.

The initial search resulted in the retrieval of 4055 records. After removing duplicates, 2898 (71.5%) records remained. After screening titles and abstracts, 2752 records (95%) were excluded, and the remaining 146 (5%) records were assessed for eligibility through full-text review. An additional 3 records were obtained through a snowball search of the reference lists in the included full-text papers. Of these 149 records, 115 (77.2%) were excluded for reasons shown in Figure 1, resulting in 32 (21.5%) papers being included in the final scoping review. Figure 1 shows the PRISMA-ScR (Preferred Reporting Item for Systematic Reviews and Meta-Analyses Extension for Scoping Reviews) flow.

### Research Question 1: Study Characteristics

**Figure 2:**
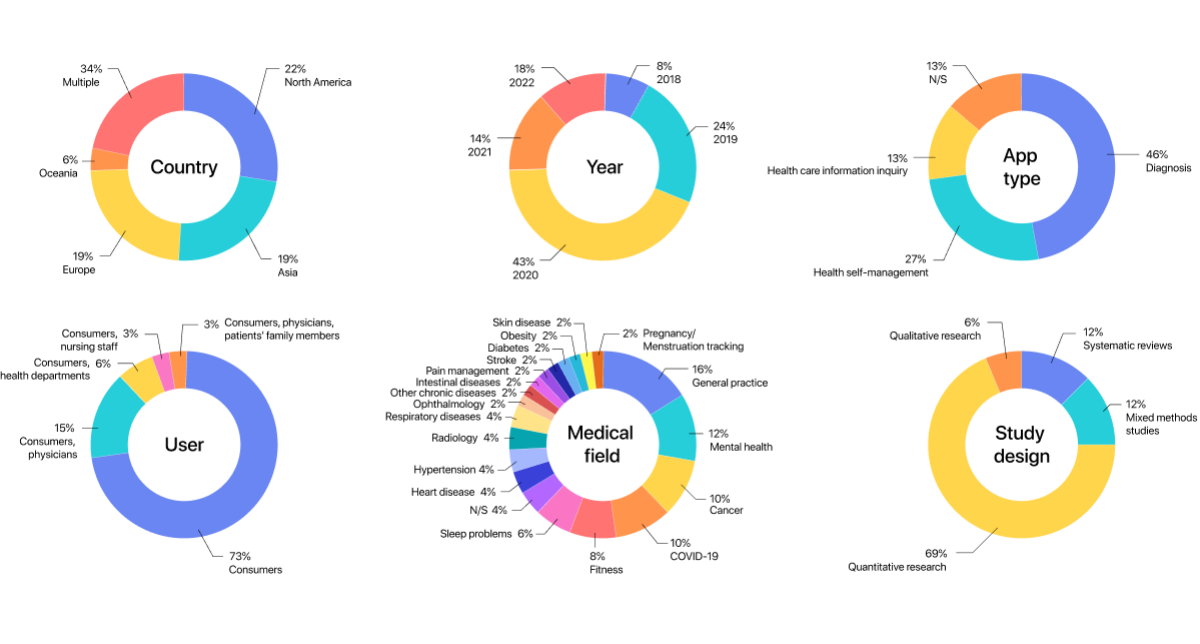
Study characteristics of DTC health care AI apps. *A single study may correspond to many items within the categories of app type, user, and medical field. Therefore, the chart percentages in the figure, which have been normalized, may differ from those in the paper. Additionally, the chart percentages did not add up to 100% due to rounding. AI: artificial intelligence; DTC: direct to consumer; N/S: not specified.

An overview of the 32 papers included in the scoping review is provided in Tables 2-4, including author, publication year, country, publication type, study objective, study design, medical field, app type, and user. We did not restrict the search year intentionally, as most health care AI review papers do [31-33]. However, the results indicated that the reviewed papers were fairly recent, with all the 32 (100%) included studies published between 2018 and 2023. Papers were from North America (7/32, 22%) [10,13,15,34-38], Asia (6/32, 19%) [39-44], Europe (6/32, 19%) [12,45-49], and Oceania (2/32, 6%) [17,50]. In addition, multiple regional cooperation was also prevalent (11/32, 34%) [51-61]. Publication types included 23 (72%) journal papers (Tables 2 and 3) [10,12,15,17,34,37,39,41,43,45-49,52-62] and 9 (28%) conference papers (Table 4) [13,35,36,38,40,42,44,50,51]. Study designs included quantitative research (22/32, 69%) [12,13,15,34,37,39,40,42-44,47-52,54,55,57-59,61], qualitative research (2/32, 6%) [35,60], and mixed methods studies (4/32, 12%) [38,41,45,46], in addition to systematic reviews (4/32, 12%) [17,36,53,56]. Most studies chose general practice (8/32, 25%) [34,37,40,41,46,49,54,55] as the target medical field. The app types mentioned in the studies included diagnosis (apps make determinations about the cause of a disease or pathology based on information provided by consumers; 14/32, 44%) [12,38,40-42,47,48,51,52,54,55,57,60,61], health self-management (apps encourage consumers to take actions to manage their continuous health status and quality of life, often in the management of chronic diseases or health problems; 8/32, 25%) [13,43,44,49,50,56,58,59], and health care information inquiry (apps extract relevant information from a large amount of health care information and generate answers based on consumer questions in common forms, such as conversational agents; 4/32, 13%) [35,37,39,46]. There were also review papers (4/32, 13%) [17,36,53,56] that reviewed apps involving more than 1 of the aforementioned function types. Some of these apps were aimed at the single-consumer group (24/32, 75%) [12,13,15,34-43,45-48,50,51,54-57,60,61], while other apps not only targeted consumers as the main users but also targeted user groups with other identities, including physicians (5/32, 16%) [17,49,52,53,59], health departments (2/32, 6%) [42,44], nursing staff (1/32, 3%) [58], and patients’ family members (1/32, 3%) [59]. Figure 2 shows an overview of the study characteristics of DTC health care AI apps, including country, year, application type, user, medical field, and study design.

**Table 2:** Overview of journal papers 1-11 included in the scoping review.

Author, country	Study objective	Study design	Medical field	App type	User
Almalki [39], Saudi Arabia	Conduct an online survey to investigate factors that influence consumers’ willingness to use COVID-19 health chatbots, as well as individual differences, the likelihood of future use, and challenges and barriers that affect their motivation.	Quantitative research: questionnaire	COVID-19	Health care information inquiry	Consumers
Cirkovic [12], Germany	Determine whether the algorithms of the 4 ophthalmic self-diagnosis apps selected from the literature change over time, as well as their efficiency of diagnostic and treatment recommendations at 3 emergency levels of diagnostic outcomes.	Quantitative research: follow-up study—a long-term research project examining the degree to which effects seen shortly after the imposition of an intervention persist over time	Ophthalmology	Diagnosis	Consumers
Demner-Fushman et al [34], the United States	Develop an online consumer health question-and-answer system that provides reliable and patient-oriented answers to consumer health queries.	Quantitative research: case analysis	General practice	Diagnosis, health care information inquiry	Consumers
Esmaeilzadeh [15], the United States	Investigate the perceived benefits and risks of AI^a^ medical devices with clinical decision support functions from the consumers’ perspective and develop models based on value perception.	Quantitative research: online survey	N/S^b^	N/S	Consumers
He et al [41], China	Develop a user needs library in the medical XAI^c^ field and design and evaluate a consumer ECG^d^ self-diagnosis system based on the needs library.	Mixed methods study: systematic review, questionnaire, interview	General practice, ECG diagnosis	Diagnosis	Consumers
Kyung and Kwon [43], Singapore	Investigate individuals’ acceptance of AI-based preventive health interventions and changes in health behaviors compliance.	Quantitative research: questionnaire, experiment	Fitness	Health self-management	Consumers
Nadarzynski et al [46], the United Kingdom	Explore the acceptability of AI-powered health chatbots in order to identify potential barriers and enablers that could have an impact on these new types of services.	Mixed methods study: interview, questionnaire	General practice	Health care information inquiry	Consumers
Ponomarchuk et al [47], Russia	Propose a machine learning method for the rapid detection of COVID-19 using cough recordings from consumer devices and develop and deploy a mobile app for COVID-19 detection using symptom checkers and voice, breathing, and cough signals.	Quantitative research: case analysis	COVID-19	Diagnosis	Consumers
Savery et al [37], the United States	Build a question-driven and natural language automated summary data set that responds to consumers’ health inquiries.	Quantitative research: experiment	General practice	Health care information inquiry	Consumers
Scott et al [17], Australia	Determine the attitudes of physicians, consumers, administrators, researchers, regulators, and industry toward the use of AI in health care.	Systematic review	N/S	N/S	Consumers, physicians
Van Bussel et al [45], the Netherlands	Through interviews with former cancer patients and physicians, expand the unified theory of acceptance and use of technology (UTAUT) model to identify the key factors driving virtual assistant acceptance among patients with cancer.	Mixed methods study: interview, questionnaire	Cancer	Diagnosis, health self-management, health care information inquiry	Consumers

^a^AI: artificial intelligence.

^b^N/S: not specified.

^c^XAI: explainable artificial intelligence.

^d^ECG: electrocardiogram.

**Table 3:** Overview of journal papers 12-23 included in the scoping review.

Author, country	Study objective	Study design	Medical field	App type	User
Da Silva et al [59], Brazil and Germany	Describe a system designed to enhance hypertensive patients’ treatment compliance.	Quantitative research: experiment	Hypertension	Health self-management	Consumers, physicians, patients’ family members
De Carvalho et al [52], the Netherlands and Romania	Review the development process of a smartphone app for skin cancer risk assessment.	Quantitative research: retrospective study	Skin cancer	Diagnosis	Consumers, physicians
Denecke et al [53], Switzerland, Norway, New Zealand, the United Kingdom, Australia, and Spain	Investigate how AI^a^ is affecting the field of participatory health and which AI apps exist in the field from a patient’s and a clinician’s perspective.	Systematic review	Diabetes, pain management, hypertension, cancer, intestinal diseases, mental health, respiratory diseases, other chronic diseases	Diagnosis, health self-management, health care information inquiry	Consumers, physicians
Fan et al [54], China, Canada, and the United States	Investigate how an AI-driven health chatbot that is extensively deployed in China can be used in the real world, what problems and barriers exist in its use, and how the user experience can be improved.	Quantitative research: case analysis	General practice	Diagnosis	Consumers
Koren et al [55], Israel and the United States	Develop and evaluate an algorithmic tool that provides symptom information to the public and their physicians to aid in decision-making.	Quantitative research: case analysis	General practice	Diagnosis	Consumers
Lau and Staccini [56], Australia and France	Examine how AI methods are presently being used by patients and consumers, present representative papers in 2018, and highlight untapped opportunities in AI research for patients and consumers.	Systematic review	Depression, mental disease, breast cancer, mental health	Health self-management	Consumers
Romero et al [48], the United Kingdom	Screen for obstructive sleep apnea based on the analysis of sleep breathing sounds recorded by consumers using smartphones at home.	Quantitative research: experiment	Obstructive sleep apnea screening	Diagnosis	Consumers
Sangers et al [57], the Netherlands and the United States	Examine the diagnostic accuracy of dermatology mobile health (mHealth) apps currently approved for consumer use in Europe, Australia, and New Zealand for the detection of precancerous and malignant skin lesions.	Quantitative research: experiment	Skin cancer	Diagnosis	Consumers
Sefa-Yeboah et al [58], Ghana and the United States	Propose an AI-based app powered by a genetic algorithm to help users with obesity self-management.	Quantitative research: experiment	Obesity	Health self-management	Consumers, nursing staff
Tschanz et al [49], Switzerland	Introduce an electronic medication management assistant to remind patients to take medication, record compliance data, inform patients of the importance of medication compliance, and provide health care teams with patients’ up-to-date medication data.	Quantitative research: case analysis	General practice	Health self-management	Consumers, physicians
Zhang et al [60], the United States and China	Investigate patients’ perceptions and acceptance of the use of AI to explain radiology reports.	Qualitative research: interview	Radiology	Diagnosis	Consumers
Zhang et al [61], the United States and China	Evaluate the effect of different AI explanations on consumer perceptions of AI-powered health care systems.	Quantitative research: experiment	Radiology	Diagnosis	Consumers

^a^AI: artificial intelligence.

**Table 4:** Overview of conference papers (n=9) included in the scoping review.

Author, country	Study objective	Study design	Medical field	App type	User
Ameko et al [13], the United States	Develop a treatment recommendation system for emotion regulation using data from participants with high social anxiety to evaluate the effectiveness of emotion regulation strategies.	Quantitative research: experiment	Emotion regulation	Health self-management	Consumers
Baldauf et al [51], Switzerland and Austria	Conduct an online survey to investigate consumers’ overall willingness to use, trust factors, and desired characteristics for 4 types of AI^a^-powered self-diagnosis apps with different data collection and processing methods.	Quantitative research: questionnaire	Skin disease, pneumonia, heart disease, sleep problems	Diagnosis	Consumers
Gupta et al [40], India	Develop a prediagnosis system that predicts potential diseases based on a patient’s symptoms and physical measurements.	Quantitative research: case analysis	General practice	Diagnosis	Consumers
Iqbal et al [42], India	Propose a new AI-based model for active surveillance of COVID-19.	Quantitative research: case analysis	COVID-19	Diagnosis	Consumers, health departments
Oniani et al [35], the United States	Use a language model to automatically answer COVID-19–related queries and conduct qualitative evaluations.	Qualitative research: expert assessment	COVID-19	Health care information inquiry	Consumers
Park et al [44], Korea	Develop a real-time monitoring system for stroke attacks based on Internet of Things sensors and machine learning technology.	Quantitative research: case analysis	Stroke	Health self-management	Consumers, health departments
Su et al [36], the United States	Examine how AI is explained in the descriptions of 40 prevalent mobile health (mHealth) apps that claim to use AI, as well as how consumers perceive these apps.	Systematic review	Fitness, mental health, meditation and sleep, nutrition and diet, pregnancy or menstruation tracking	Diagnosis, health self-management, health care information inquiry	Consumers
Sellak et al [50], Australia	Design a model aimed at understanding how to design digital health interventions that can change lives, as well as which software design components enhance consumers’ acceptance, adherence, and sustained engagement.	Quantitative research: case analysis	Fitness	Health self-management	Consumers
Tsai et al [38], the United States	Examine how explanations can be used to improve the diagnostic transparency of online symptom checkers.	Mixed methods study: interview, experiment, questionnaire	COVID-19	Diagnosis	Consumers

^a^AI: artificial intelligence.

### Research Question 2: Barriers

**Figure 3:**
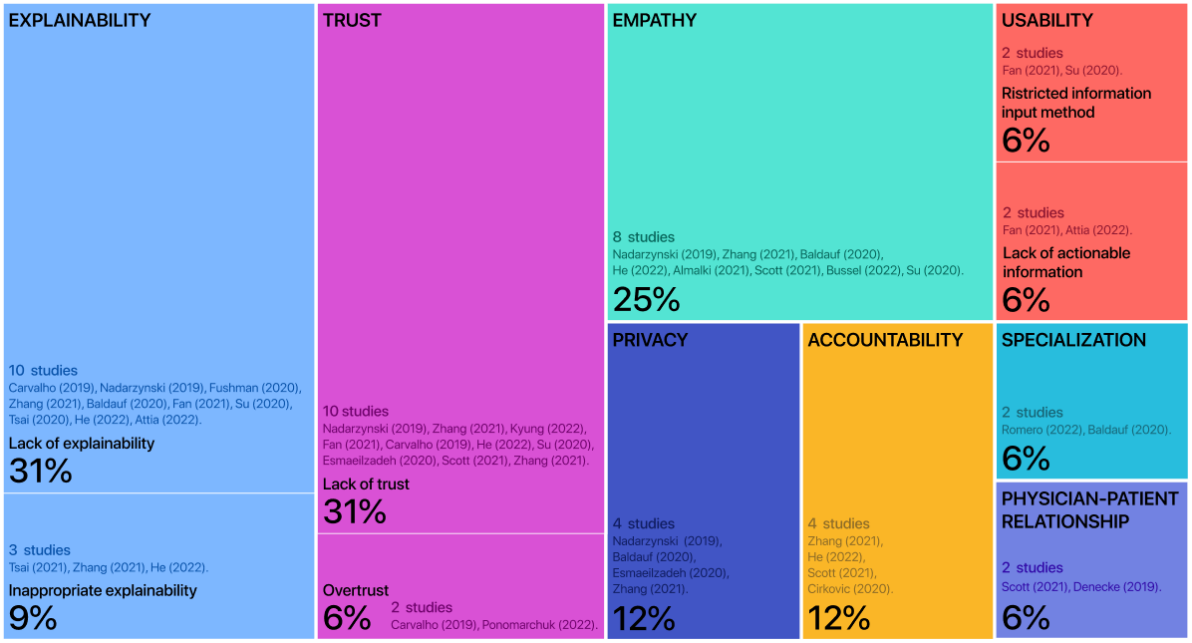
Existing barriers faced by DTC health care AI apps, along with their subthemes, and the number of studies mentioning them. *The chart percentages in the figure correspond to the percentages in the paper. AI: artificial intelligence; DTC: direct to consumer.

We identified 8 barriers to designing and developing DTC health care AI apps: (1) lack of explainability and inappropriate explainability, (2) lack of empathy, (3) effect of information input method and content on usability, (4) concerns about the privacy protection ability, (5) concerns about the AI accountability system, (6) lack of trust and overtrust, (7) concerns about specialization, and (8) the unpredictable future physician-patient relationship. These 8 existing barriers faced by DTC health care AI apps, along with their related subthemes, and the number of studies mentioning them are shown in Figure 3.

#### Explainability

##### Lack of Explainability

Of the 32 studies, 10 (31%) [10,34,36,38,41,46,51,52,54,60] pointed out that the explanations provided by existing DTC health care AI apps are insufficient. Existing studies mostly provided explanations primarily for domain experts, paying less attention to the explainability needs of lay users, such as consumers [41]. In addition, 2 (6%) studies [46,51] pointed out that current DTC health care AI apps lack the explanations of relevant knowledge in the AI field (ie, the explanations of the working principle of the machine learning algorithm used by the apps, such as how AI correctly responds to consumers’ health consultations [46]). Furthermore, 4 (13%) studies [34,46,51,54] indicated that current DTC health care AI apps lack explanations of relevant knowledge of the medical field, such as highly specialized medical terminology [34] and rare diseases that have only been discussed in professional literatures [54], and 4 (13%) studies [36,38,51,60] pointed out the disadvantages of a lack of explainability, which caused consumers to doubt the usefulness, accuracy, and safety of the apps and even possibly view them as a threat. Moreover, 1 (3%) study [51] mentioned the advantages of providing explanations, which aided consumers in understanding the reasoning of the system, and this understanding was crucial for boosting the trust of lay users.

##### Inappropriate Explainability

Of the 32 studies, 3 (9%) [38,41,60] highlighted that current DTC health care AI apps contain inappropriate explanations. Specifically, 2 (6%) studies [38,41] mentioned that excessive explanations can result in information overload for users, which in turn would negatively impact the user experience and might cause users to ignore system prompts or suggestions. In addition, 1 (3%) study [60] pointed out that the poor information quality of explanations would be considered by users as “invalid, meaningless, not legit, or a bunch of crap” and even cause users to perceive it as a risk, prompting them to seek secondary confirmation of information through other channels (eg, online search or consultation with a doctor) to ensure their own safety. Furthermore, 2 (6%) studies [38,41] indicated that improper levels of transparency or inappropriate presentation formats in explanations can pose risks, potentially harming the interests of other stakeholders in the AI system or affecting the authenticity of users’ future performances. Specifically, inappropriate transparency of explanations might lead to the disclosure of sensitive details and intrusion of systems, harming the interests of AI service providers and violating the privacy of other consumers [38]. Explaining to users how a particular feature would accurately affect the disease diagnosis might affect their performance authenticity in the future diagnosis of related diseases, allowing them to manipulate the likelihood of being diagnosed or not diagnosed by deliberately meeting or avoiding meeting the characteristic threshold, respectively [41]. Inappropriate presentation forms of explanations, such as the function of counterfactual explanations that allowed users to freely edit data to view different diagnostic results, were popular with physicians because they met the needs of medical users to test different data and corresponding diagnostic possibilities, but they might become technical loopholes in the commercialization of DTC health care AI apps. Users could exploit this feature to input data for multiple individuals and view different results, thereby avoiding multiple payments and compromising the economic interests of the AI service provider [41].

#### Empathy

In a total of 8 (25%) studies [17,36,39,41,45,46,51,60], users felt that AI lacked empathy and was impersonal. Among them, users in 2 (6%) studies [45,46] felt that AI was unable to understand emotion-related issues, especially mental health problems, and 2 (6%) studies [41,60] pointed out that the information-conveying method of AI, such as transmitting complex disease information without human presence [60] and explaining the disease from the perspective of “how bad it is” [41], could also lead users to think that AI is indifferent and inhumane. In addition, 5 (16%) studies [36,39,41,46,60] reported that the lack of empathy would lead to a series of negative consequences, including triggering users’ frustration, disappointment, anxiety, and other negative emotions [36,60]; impeding users’ acceptance of such apps [39,46]; and even affecting their subsequent treatments [41]. Furthermore, according to 2 (6%) studies [46,51], some users preferred to consult human physicians rather than AI because they could offer comfort and spiritual support.

#### Usability

##### Restricted Information Input Method

Of the 32 studies, 2 (6%) [36,54] pointed out that the restricted information input method in DTC health care AI apps (eg, a single way of typing) made users feel helpless and frustrated, which was contrary to their usage expectations, and even made them inclined to discontinue use.

##### Lack of Actionable Information

Of the 32 studies, 2 (6%) [10,54] pointed out that DTC health care AI apps lacked actionable information content, failing to inform users of the next actions to take, such as where to seek medical assistance.

#### Privacy

In total, 4 (12%) studies [15,46,51,60] raised concerns about the ability of DTC health care AI apps to protect privacy, such as safeguarding users’ sensitive health-related information from data breaches. Users were concerned that their personal information (eg, habits, preferences, and health records) would be collected without their knowledge [46], that anonymous data would be re-identified through AI processes [15], that data would be sold by companies for secondary exploitation [51], and that their health data would be hacked and used against them [60].

#### Accountability and Supervision

In total, 4 (12%) studies [12,17,41,60] raised concerns about the accountability of DTC health care AI apps, and 2 (50%) of these studies [17,41] indicated that only few controversial studies exist on the distribution of AI responsibilities. Another study [12] exemplified the practice of some application manufactures who made general recommendations (eg, “recommend emergency care”) for almost every diagnosis, thereby transferring responsibility to users. In some countries, according to 1 (3%) study [17], there were concerns with the supervision of DTC health care AI apps. The absence of human supervision during the design, development, and deployment of AI not only failed to ensure the anticipated benefits but also posed a risk of potential injury to users.

#### Trust

##### Lack of Trust

A total of 10 (31%) studies [15,17,36,41,43,46,52,54,60,61] pointed out that users lacked trust in DTC health care AI apps. Among them, 5 (50%) studies [15,17,54,60,61] distrusted AI due to inadequate performance or the lack of performance explanations, 3 (30%) studies [41,43,46] found that even if the AI performed as well as or better than human physicians, users still placed more trust and reliance on humans, and 3 (30%) studies [15,36,52] indicated that users’ lack of trust might cause them to disregard AI recommendations or even stop using such apps.

##### Overtrust

Based on the calibration between trust and competence, trust can be divided into 3 levels: calibrated trust, distrust, and overtrust. Distrust refers to users being less willing to trust AI compared to similar human providers, even if AI shows superior performance; overtrust refers to the user’s trust in the system beyond its actual capabilities [63]. Of the 32 studies, 2 (6%) [47,52] indicated that users’ overtrust issues in DTC health care AI apps would impose a double burden on both individuals [47,52] and society [47]. At the individual level, 2 (6%) studies [47,52] pointed out that overtrusting false-positive results could result in users’ negative emotions (eg, stress [52]). Tools with a high rate of false positives might also reduce users’ trust in true-positive results [47]. In addition, 1 (3%) study [52] pointed out that overtrusting false-positive results could trigger users’ unnecessary behaviors, such as unnecessary medical treatment, while 1 (3%) study [47] pointed out that overtrusting false-negative results would provide users with a false sense of security and delay the disease diagnosis. At the societal level, 1 (3%) study [47] indicated that individuals’ overtrust in false-positive results could overwhelm the entire health care system, whereas individuals’ overtrust in false-negative results could exacerbate the social transmission of diseases (eg, COVID-19).

#### Specialization

In total, 2 (6%) studies [48,51] raised concerns about the specialization of DTC health care AI apps. To be specific, users in 1 (3%) study [51] doubted the feasibility of substituting consumer-grade equipment for professional medical-grade equipment. For example, they argued that an artificial intelligence–electrocardiogram (AI-ECG) smartwatch that measured only the wrist could not replace a traditional ECG machine with 12 electrodes for detecting heart diseases. The other study [48] pointed out that the professional effect of DTC health care AI apps is influenced by the using environment. For example, an app that detects obstructive sleep apnea, which is affected by background noise, might work in tightly controlled laboratory conditions but might not be as accurate in in-home environments.

#### Physician-Patient Relationship

In total, 2 (6%) studies [17,53] believed that DTC health care AI apps would make the physician-patient relationship less predictable. As a result of AI user empowerment and the emergence of “do-it-yourself” medicine, users were less reliant on medical experts [17] and expert medical advice [53]. The effects of AI on the physician-patient relationship remains to be evaluated by more studies [53].

### Research Question 3: Design Recommendations

**Figure 4:**
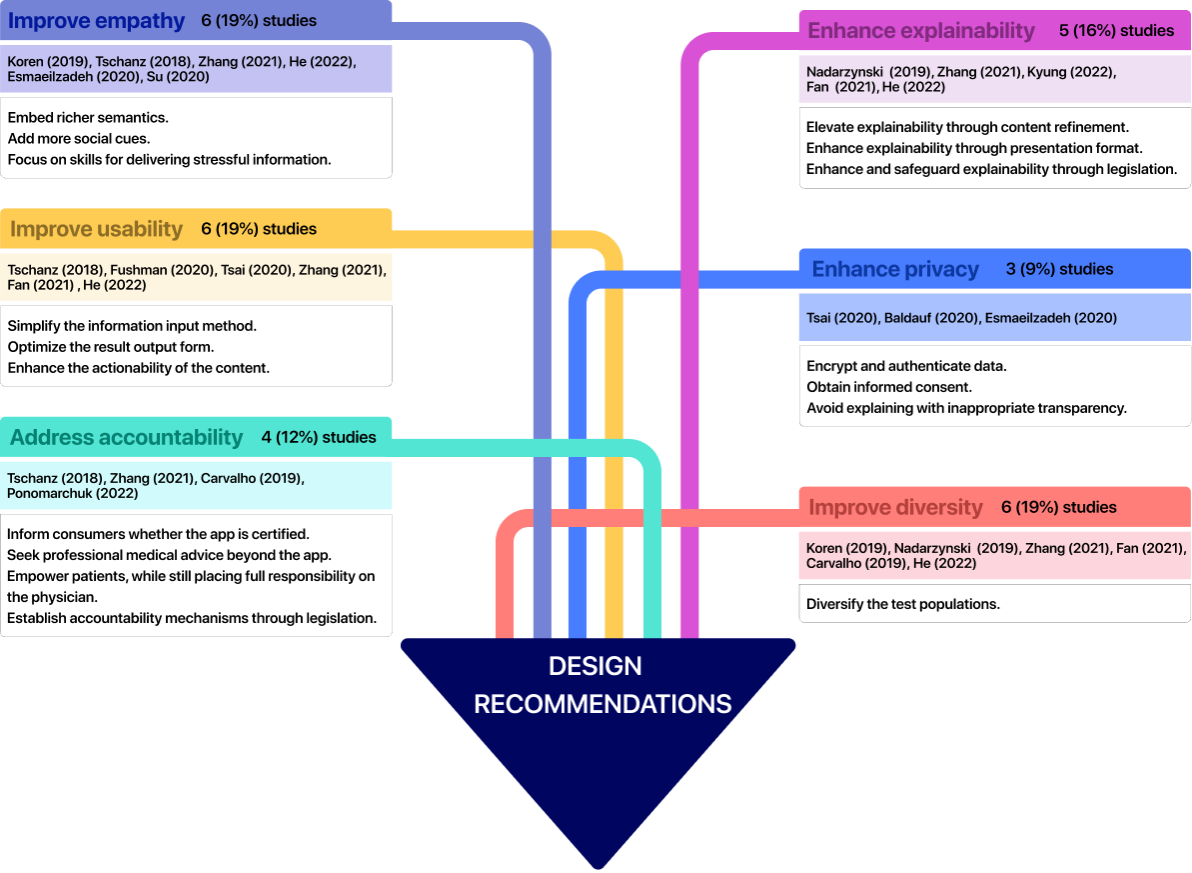
Future design recommendations of DTC health care AI apps, along with their subthemes and the number of studies mentioning them. AI: artificial intelligence; DTC: direct to consumer.

The themes of design recommendations covered 6 types of recommendations and their specific contents mentioned by existing studies when designing and developing DTC health care AI apps: (1) enhance explainability, (2) improve empathy, (3) improve usability, (4) enhance privacy protection ability, (5) address AI accountability at both the individual and the government level, and (6) improve the diversity of participants to enhance inclusion. These 6 design recommendations for DTC health care AI apps, as well as the related subthemes and the number of studies mentioning them, are shown in Figure 4.

#### Enhance Explainability

Of the 32 studies, 5 (16%) [41,43,46,54,60] suggested designing and developing explainable DTC health care AI apps from 3 perspectives: the explanations’ primary content, their presentation form, and their legislation. First, 4 (13%) studies [41,46,54,60] provided content recommendations for explanations: input (explanations of the input data) [41,54], output (explanations of the generated output) [41], the how (explanations of how the system as a whole works) [41,54,60], performance (explanations of the capabilities, limitations, and verification process of the current system) [41,46,54,60], the why (explanations as to why, and why not, the system made a specific decision) [41], what-if (explanations to speculate on the system’s output under a particular set of settings and to describe what the system would do) [41], responsibility (explanations of the system’s accountability) [41], ethics (explanations of information from regulatory approvals or peer-reviewed publications that validated the system) [41], the social effect (explanations of the results of other social subjects using the system) [41], and domain knowledge (explanations of specific AI or medical terms and information sources in the system) [41,54]. Second, based on the complex diversity of consumer groups with varying domain knowledge, cognitive styles, and urgency of symptoms, 1 (3%) study [41] provided suggestions for explanations’ presentation forms: using a progressive disclosure approach to present various levels and formats of explanations to meet the needs of a wider consumer group. Third, 1 (3%) study [43] provided legislative suggestions for explanations: future governments and regulatory agencies, particularly in the medical field, would need to further establish and improve the legal framework for transparent AI to safeguard the right of consumers to obtain explanations based on algorithmic decisions.

#### Improve Empathy

In total, 6 (19%) studies [15,36,41,49,55,60] designed and developed empathetic DTC health care AI apps. Specifically, 3 (9%) studies [15,36,49] suggested that such apps could directly incorporate conversational agents or refer to research results in this field to embed richer semantics [49] and add more social cues [15], while 2 (6%) studies [41,60] suggested focusing on skills for delivering stressful information.

#### Improve Usability

In total, 6 (19%) studies [34,38,41,49,54,60] enhanced the usability of DTC health care AI apps in 3 aspects: information input method, result output form, and content actionability. Concerning the information input method, 1 (3%) study [54] suggested simplifying the way consumers input data (eg, by sharing and describing information in the form of audio recordings) to save their time and effort, while 1 (3%) study [49] simplified the way consumers input data (eg, by barcode-scanning prescription data) to reduce the risk of manual data entry errors. Concerning the result output form, 1 (3%) study [34] translated or simplified highly specialized language that was difficult for consumers to understand (eg, rare diseases that were only discussed in professional literature) and also provided illustrations to summarize the output; 2 (6%) studies [38,41] suggested avoiding outputting too much and too detailed information at once so as to prevent consumers from information overload. Concerning content actionability, 1 (3%) study [54] suggested, at the initial stage of interaction, providing introductory materials to teach consumers the most effective way to use advanced technology (eg, introducing basic functions, limitations, and the use process); 1 (3%) study [41] suggested, during the interaction, clearly explaining the purpose of the current operation and context-related information to consumers and informing them of the results of the current operation directly on the interface; and 1 (3%) study [54] suggested, at the end of the interaction, informing consumers of the next step (eg, where to seek medical help).

#### Enhance Privacy

Of the 32 studies, 3 (9%) [15,38,51] suggested enhancing the privacy protection capabilities of DTC health care AI apps to prevent consumers’ privacy from being violated. Specifically, the recommended using state-of-the-art technology to encrypt and authenticate users’ health data [51], obtaining informed consent for health care purposes to prevent data from being resold and exploited [15], and avoiding explanations with inappropriate transparency (eg, leaking flaws in algorithms or detecting sensitive data sources) to prevent systems from being intruded [38].

#### Address Accountability

In total, 4 (12%) studies [43,45,48,56] addressed the accountability issues of DTC health care AI apps from both individual and government perspectives. At the individual level, 1 (3%) study [47] addressed accountability by informing consumers whether the app was officially certified and encouraging them to seek professional medical advice or clinical testing beyond the app, and 1 (3%) study [49] empowered patients and provided them with more responsibilities (eg, motivating patients to take their medications, while informing them of possible drug interactions) but still opted for human medical staff to undertake the responsibility for complete drug therapy. At the government level, 1 (3%) study [60] suggested developing policies or guidelines to regulate the use of such apps and establish accountability mechanisms through legislation for AI output, and 1 (3%) study [52] suggested that national health authorities should clarify the position of these apps in the health care system (eg, whether they were for laypersons, general practitioners, or specialists).

#### Improve Diversity

In total, 6 (19%) studies [41,46,52,54,55,60] designed and developed DTC health care AI apps by diversifying the test populations of the diseases targeted by apps in the future. Specifically, studies focused on clinical populations [46], community populations [46], marginalized populations (eg, populations with low education levels [60] and the elderly [54,60]), and children [55] and the cultural and social factors in these populations [54] in order to capture more diverse user needs and develop a more comprehensive solution.

## Discussion

### Principal Findings

In the context of a surge in DTC health care AI apps, this scoping review identified 32 studies in the existing academic literature that address this topic. The review summarized the characteristics of existing studies on DTC health care AI apps, highlighted 8 categories of extant barriers, and pointed out 6 categories of design recommendations.

### Study Characteristics

In terms of the developmental timeline, although AI has been extensively used across various sectors of health care, studies focusing on DTC health care AI apps are still in their nascent stages. We did not artificially restrict the time frame for our review; however, the papers included in our results were all published recently (between 2018 and 2023).

In terms of geographical origins, the studies on DTC health care AI apps predominantly came from high-income countries, particularly the United States. This aligns with other reviews in the domain of health care AI [21,31,64]. This correlation is intrinsically tied to the fact that a more advanced digital health care infrastructure (eg, electronic health records (EHRs), health information exchanges (HIEs), and telehealth platforms) is present in these countries. More geographically diverse research is needed in the future, and we particularly expect a surge in studies originating from LMICs, because AI is considered a technology that can help bridge the digital gap and reduce health inequities worldwide [2,3,64]. However, the current study outcomes from high-income countries cannot be directly transferred to low-income regions due to significant risks, such as output bias, poor performance, or erroneous results, when using AI solutions trained in contexts that differ substantially from the local populations [65]. When AI systems are applied to new populations with differing living environments or cultural backgrounds, adaptations to the local clinical settings and practices are required, and the measures and outcomes for design, development, and evaluation may vary [41,66].

In terms of the study design, the majority of the papers we reviewed opted for quantitative methods to evaluate the apps, such as collecting performance metrics when consumers use the apps or obtaining quantitative data on existing user experience dimensions through questionnaires. Fewer papers delved into the barriers and recommendations arising from users’ usage of DTC health care AI apps. However, given that the emergence of such apps is still a nascent phenomenon, future work requires more qualitative research to explore the effects generated by these technological systems when used in society, to dig out initially overlooked new themes or deeper insights, and to assess user experiences beyond what short-term metrics can capture, while also incorporating edge cases that large-scale studies may overlook [67,68].

In terms of medical fields, existing studies on DTC health care AI apps primarily focused on the field of general practice. This is understandable because general practice usually serves as the first medical contact point for patients [69], thereby having a broad spectrum of user needs. Moreover, the health issues diagnosed and treated in general practice are generally more common and less complex [70], thereby presenting relatively lower risks. Consequently, most studies chose general practice as the entry point for the medical fields of designing and developing DTC health care AI apps.

In terms of intended users and provided functionalities among studies on DTC health care AI apps, some were designed solely for single-consumer user groups, offering functions such as disease diagnosis, health self-management, and health care information inquiry. Others also connected with other user groups, including physicians, family members, nursing staff, and health care departments, generally to alert these groups to abnormal conditions of consumer users. For example, these functionalities may include alerting hospitals about consumer user falls due to stroke, notifying physicians and family members about medication adherence issues, referring users with high-risk skin cancer ratings to doctors, or informing health care departments about potential diagnoses of COVID-19 or other infectious diseases. However, it is crucial to note that although such intelligent functionalities for alerting other groups about users’ anomalies may contribute positively to users’ health and the efficient functioning of health care systems, they also pose risks related to consumers’ human rights, democracy, false positives due to erroneous data capture, and even the manipulation of users with low behavioral capacity [71]. Future DTC health care AI apps, when designing features that involve 2 or more user groups, must consider how to allocate, balance, and constrain power among various stakeholders, while simultaneously ensuring ethical and legal compliance as they seek to benefit consumer groups in need.

### Barriers and Design Recommendations

In terms of barriers and design recommendations, it is noteworthy that many challenges are not confined solely to apps targeting consumers; rather, they exhibit considerable similarities with the issues encountered by health care AI systems designed for other user groups, such as health care professionals. First, privacy concerns have been widely recognized as a significant barrier to the application of AI in the health care domain [20,21,72,73]. Privacy protection has become a hot topic in the health care AI research field [74], with numerous studies dedicated to developing innovative privacy-preserving solutions without compromising the performance of big data–driven AI models. These include developing privacy-enhancing technologies, such as homomorphic encryption [75], securing multiparty computation and differential privacy [76], and exploring new training methods and data governance models, such as distributed federated machine learning using synthesized data from multiple organizations [77], data-sharing pools [78], data trusts [79], and data cooperatives [80]. Second, the lack of clarity in accountability and regulation has also been universally identified in prior research as a key obstacle to the application of AI in health care [81-83]. Despite the existence of various worldwide policies and regulations concerning AI accountability and regulation, such as WHO [84], the General Data Protection Regulation (GDPR) [85], the Food and Drug Administration (FDA) [86], Health Canada [87], and the AI Act [88], the rapid advancement of AI technology makes it difficult for existing regulatory frameworks to keep up, let alone be able to anticipate its potential risks and impacts. Taking the AI Act, which is currently being advanced in Europe, as an example, the emergence of new generative AI systems, such as ChatGPT, has already posed challenges to the universality and applicability of this legislation [89]. Furthermore, usability has also been shown in previous studies concerning physicians as an aspect that doctors wish to see improved in health care AI tools, such as clinical decision support systems [41,66]. Additionally, the evolution of physician-patient relationships has been identified as a key point requiring long-term tracking following the deployment of various types of health care AI systems [90].

In addition to identifying challenges similar to those faced by health care AI systems targeted at other user groups, this review further identified some more subtle obstacles that are particularly worth noting in consumer-facing systems and distilled corresponding design recommendations, including enhancing human-centered explainability, establishing calibrated trust and addressing overtrust, demonstrating empathy in AI, improving the specialization of consumer-grade products, and expanding the diversity of the test population.

#### Enhance Human-Centered Explainability

The review findings identified current barriers to explainability in DTC health care AI apps, which included not only providing inadequate explanations to consumers (a lack of explanations relating to both AI and medical domain knowledge) but also providing inappropriate explanations to consumers (excessive content caused information overload to consumers, low-quality content exposed consumers to risks and burdens, and improper transparency and presentation forms could adversely impact other stakeholders’ interests in the system). To address these barriers, our review offered design recommendations for improvements in the content, form, and legislative aspects of explanations, which future research can consider.

Furthermore, we believe that the review results demonstrate and re-emphasize the importance of designing, developing, and evaluating AI explainability from a human-centered perspective. As AI increasingly powers decision-making in high-risk areas, such as health care, explainable artificial intelligence (XAI), aimed at enabling humans to understand the logic and outcomes of AI systems, has become a research hotspot in recent years [91-95]. Within this interdisciplinary field, algorithm-centered approaches aim to enhance the transparency of AI models and to develop inherently explainable models [96], while human-centered approaches emphasize considerations such as who the users of explanations are, why explanations are needed (eg, how social and individual factors influence explainability objectives), and what the timing and context of providing explanations (eg, contextual variations in explainability across different application domains) are [97,98]. As shown in our findings, consumers of health care AI had various needs concerning the content and form of explanations, and their interactions with explanations could influence their adoption toward the apps and subsequent behavior. Furthermore, wrong explanation design could produce correlation effects on other stakeholders in the AI system. All these findings indicate that the challenges in explainability in DTC health care AI apps are not merely technical issues concerning algorithmic transparency but also significantly involve human factors. Future studies need to enhance the explainability of DTC health care AI apps from a human-centered perspective, focusing on the cognitive abilities, physical characteristics, and social and psychological factors of the human in the loop, as well as how these human factors interact with explanations, AI systems, and the environment. This will enable the design of DTC health care AI apps that meet user needs and enhance human performance, safety, and overall well-being.

#### Establish Calibrated Trust and Pay Special Attention to Overtrust

Our findings indicated that current DTC health care AI apps face challenges related to trust, including both a lack of trust and overtrust. The need to establish calibrated trust in AI systems, meaning cultivating the users’ ability to know when to trust (accept correct advice) or not trust (reject erroneous advice) AI [99], has reached a consensus in current research [100]. Under this premise, we believe that future designs of DTC AI apps should pay more attention to the issue of overtrust. There are multiple rationales for this focus. On the one hand, from an academic research perspective, most extant studies on AI trust predominantly center on enhancing users’ trust [101-104], with less attention given to the issue of overtrust; on the other hand, from a practical application perspective, 3 influencing factors also need to be considered:

- First, the users’ background knowledge. Consumers often possess limited prior knowledge of both medical and AI domains related to these apps [4], affecting their receptivity to AI advice. Research has shown that domain experts are more likely to question AI suggestions, whereas nonexperts are more receptive to them [105].
- Second, the differential risk in decision-making: Consumers and health care professionals differ in their risk assessments when facing AI advice. Typical consumers are loss averse; for them, changes for the worse (losses) loom larger than equivalent changes for the better [106]. Hence, they are more inclined to accept AI advice and take subsequent medical actions, rather than potentially missing out on timely disease diagnosis and treatment if AI advice is not adopted [4]. In contrast, the biggest concern of health care professionals when adopting new products to assist medical diagnosis may not be the pursuit of improvement in work performance but the potential risks to patients’ lives and health [107], so their adopting is relatively cautious.
- Third, the drive for commercial interests may also prompt these apps to exaggerate their capabilities, thereby further exacerbating the issue of consumer overtrust [36].

Therefore, in summary, although both domain expert and nonexpert users may display overreliance on automation [108], physicians’ overtrust in AI diagnostic features is not commonly observed at this current stage of medical AI development; many reviews in the AI domain concerning physician users, while identifying trust issues, primarily discuss a lack of trust [66,109]. However, consumer overtrust in health care AI, along with the ensuing personal and societal effects, has already emerged as an issue that needs to be considered sooner rather than later.

#### Demonstrate Empathy in Artificial Intelligence

Our review indicated that even if AI can be more accurate and logical, its lack of empathy may hinder consumer acceptance of DTC health care AI apps. Empathy, defined as the ability to understand or feel what other individuals are experiencing from their frame of reference [110], is widely acknowledged as a fundamental value for achieving optimal health care practices. It is crucial for enhancing patient satisfaction, treatment compliance, and clinical outcomes [111-113]. In conventional medical settings, health care professionals act as the conveyors of empathy, while patients are the recipients [114]; in human-AI collaborative medical settings, such as physicians using AI for diagnostic assistance, AI primarily contributes to improving efficiency and decision-making quality, allowing health care professionals to have more time and energy to convey empathy and improve overall treatment satisfaction [115]; However, in DTC health care AI scenarios, the initial touchpoint no longer has a human element, necessitating AI to become the direct conveyor of empathy.

The topic of AI empathy in health care has become a research hotspot [116-118]. To address this challenge, our review offered several design recommendations: embedding richer semantics and social cues through conversational agents, as well as techniques for conveying stressful information. Current cutting-edge research supports these design suggestions for enhancing empathy through conversational agents. Studies indicate that the new generation of AI chatbots, such as ChatGPT, has scored higher than human doctors in terms of empathy [119]. Our review is current up to March 2023, and the research included in the review has not yet covered ChatGPT. Therefore, the future integration of ChatGPT or similar large language model chatbots could potentially help alleviate the empathy barriers in DTC health care AI apps.

#### Improve Specialization of Consumer-Grade Products

Concerns regarding the specialization of DTC health care AI apps are totally understandable. First, from a scientific and technological standpoint, many health care AI apps on the consumer market have scarcely undergone original research for effectiveness or are loosely based on scientific studies but lack a scientific consensus on their efficacy [120]. Furthermore, the data collection devices for these apps are often consumer-owned smartphones, personal computers, or wearables designed for portability, rather than specialized medical devices tailored for specific disease domains.

Second, in terms of regulatory frameworks, in the United States, where most companies producing DTC health care AI products are located, existing tiered regulatory systems permit the manufacture of general wellness products without adhering to regulations typically applicable to devices intended for diagnosing or treating diseases [86]. Consequently, driven by commercial interests, the current market is flooded with numerous tools that are approved as general health products but subtly imply that they can be used for diagnosis or treatment. Consumers can easily access these products, although the products may not have undergone rigorous testing and regulation, thus rendering their effectiveness uncertain [71,121].

Existing research is working to close the performance gap between consumer-grade products and clinical-grade medical devices through technological innovations, for example, developing high-precision flexible sensors to improve the data collection capabilities of wearable devices [122,123], as well as through algorithm-hardware cooptimization to ensure model quality is not compromised while achieving device miniaturization [124]. However, overcoming this barrier will require not only technological advancements but also further refinement of the approval and regulatory frameworks for consumer-grade AI products in the future.

#### Expand the Diversity of Test Populations

The need to expand the diversity of test populations is also a future direction in the design and development of DTC health care AI apps, as identified by our review. It is worth noting that whenever this theme is mentioned in the papers included in our review, it appears in the *Limitations* or *Future Work* section. This indirectly indicates that it is a prevalent yet unresolved issue in this field of research. In existing research, either the test population involves a small subset of patients in the specific disease area with limited demographic characteristics and health information literacy or it is not even the target population for the disease but rather comprises participants recruited through convenience sampling. However, if such apps truly enter the market, their actual consumer users constitute an extremely broad and heterogeneous group, with widely varying demographic characteristics, education levels, and health and information literacy [125]. Applying AI models trained on small sample data and user feedback obtained from these samples to a broader population could pose multiple risks, including inaccuracies in AI diagnostics and predictions, poor generalization ability to unseen patient data, and perpetuating biases and exclusions against marginalized groups [126]. These risks could consequently misguide clinical decisions, exacerbate health care inequalities, and trigger legal and ethical crises. Future studies on DTC health care AI apps indeed needs to consider the diversity of the consumer population in terms of culture, society, demographics, and knowledge accomplishment in order to develop more accurate and inclusive health care AI solutions.

### Limitations

This study has a few limitations. First, we retrieved papers written in English, thereby potentially overlooking influential papers published in other languages. Additionally, we only captured papers that were found in the search. Given the novelty of the field and terminology associated with DTC health care AI apps, some relevant studies may have been omitted. However, we attempted to mitigate this limitation by using Google Scholar to search for gray literature and by snowball-sampling from the reference lists of relevant papers. Due to the wide-ranging formats and scopes of gray literature, it often serves as a robust source of evidence in systematic reviews, offering extra data not found in commercial publications, thus reducing publication bias and enabling a more balanced view of evidence [27]. Google Scholar’s gray literature includes papers from databases that have not yet been formally published, such as arXiv and medRxiv, helping capture research that might be overlooked due to the novelty of the field and terminology.

Furthermore, when using qualitative thematic analysis to synthesize study findings and generate themes, the themes produced were potentially influenced by the prior research experience and personal understanding of the 3 authors. Therefore, the themes may not be entirely comprehensive or may differ when other researchers replicate the coding process. To minimize potential coding bias, we strictly adhered to the 6 key steps of qualitative thematic analysis: familiarizing oneself with the data set; coding; generating initial themes; developing and reviewing themes; refining, defining, and naming themes; and writing up. Each step underwent group discussions, triangulation, and interrater reliability checks among the 3 authors to resolve disagreements and reach a final consensus, thereby striving to maintain consistency and reduce individual differences.

### Conclusion

To the best of our knowledge, this is the first study to systematically summarize and organize academic research targeting consumers through DTC health care AI apps. In this study, we delineated the current characteristics of studies focusing on DTC health care AI apps, identified 8 existing barriers, and offered 6 design recommendations. We believe that future research, by considering the key points raised in this study, addressing existing barriers, and referencing design recommendations, can better advance the study, design, and development of DTC health care AI apps, thus improving the health care services they provide.

## Appendix

Multimedia Appendix 1 Preferred Reporting Items for Systematic Reviews and Meta-Analyses Extension for Scoping Reviews (PRISMA-ScR) checklist.

Multimedia Appendix 2 Database search details.

## Abbreviations

AI: artificial intelligence
DTC: direct to consumer
ECG: electrocardiogram
GPT: Generative Pretrained Transformer
LMIC: low- and middle-income country
PRISMA-ScR: Preferred Reporting Items for Systematic Reviews and Meta-Analyses Extension for Scoping Reviews
XAI: explainable artificial intelligence

## Data Availability

All data generated and analyzed during this study are included in this published paper and its Multimedia Appendices.
