# Supplementary material for: Existing Barriers Faced by and Future Design Recommendations for Direct-to-Consumer Health Care Artificial Intelligence Apps: Scoping Review
Source: J Med Internet Res. 2023 Dec 18;25:e50342. doi: 10.2196/50342 (PMC10758939; doi:10.2196/50342)
Supplement: Multimedia Appendix 2 [file jmir_v25i1e50342_app2.doc]

### Multimedia Appendix 2: Database search details.

Run on 27 of March 2023

| **Database** | **Where** | **Search terms** | **Number**  **of studies** |
| --- | --- | --- | --- |
| Web of Science  [LINK](https://www.webofscience.com/wos/alldb/summary/22610e75-1e89-40ed-99dd-25c4e2eae9de-7c87aae6/date-descending/1) | Topic  (TS) | TS = (("AI" OR artificial intelligence OR "ML" OR machine learning OR "DL" OR deep learning) AND (medical OR healthcare OR health) AND (consumer OR consumers) AND (application OR applications OR "App" OR "Apps" OR system OR systems OR service)) | 2,338 |
| Scopus  [LINK](https://www.scopus.com/results/results.uri?sort=plf-f&src=s&sid=7e9c7ece3b2de4e33c9fd57f1cab05ab&sot=a&sdt=a&sl=291&s=(+ABS+(+"AI"+OR+"artificial+intelligence"+OR+"ML"+OR+"machine+learning"+OR+"DL"+OR+"deep+learning"+)+AND+ABS+(+"medical"+OR+"healthcare"+OR+"health"+)+AND+ABS+(+"consumer"+OR+"consumers"+)+AND+ABS+(+"application"+OR+"applications"+OR+"App"+OR+"Apps"+OR+"system"+OR+"systems"+OR+"service"+)+)&origin=searchadvanced&editSaveSearch=&txGid=3bd8d303c17bcc86b285c3d1030f84ac) | Abstract  (ABS) | ( ABS ( "AI" OR "artificial intelligence" OR "ML" OR "machine learning" OR "DL" OR "deep learning" ) AND ABS ( "medical" OR "healthcare" OR "health" ) AND ABS ( "consumer" OR "consumers" ) AND ABS ( "application" OR "applications" OR "App" OR "Apps" OR "system" OR "systems" OR "service" ) ) | 883 |
| ACM digital library  [LINK](https://dl.acm.org/action/doSearch?fillQuickSearch=false&target=advanced&expand=dl&AllField=Abstract%3A(("AI"+OR+artificial+intelligence+OR+"ML"+OR+machine+learning+OR+"DL"+OR+deep+learning)+AND+(medical+OR+healthcare+OR+health)+AND+(consumer+OR+consumers)+AND+(application+OR+applications+OR+"App"+OR+"Apps"+OR+system+OR+systems+OR+service))&startPage=0&sortBy=EpubDate_desc) | Abstract | (("AI" OR artificial intelligence OR "ML" OR machine learning OR "DL" OR deep learning) AND (medical OR healthcare OR health) AND (consumer OR consumers) AND (application OR applications OR "App" OR "Apps" OR system OR systems OR service)) | 267 |
| IEEE Xplore  [LINK](https://ieeexplore.ieee.org/search/searchresult.jsp?action=search&matchBoolean=true&queryText=(("Abstract":"AI" OR "Abstract":"artificial intelligence" OR "Abstract":"ML" OR "Abstract":"machine learning" OR "Abstract":"DL" OR "Abstract":"deep learning") AND ("Abstract":medical OR "Abstract":healthcare OR "Abstract":health) AND ("Abstract":consumer OR "Abstract":consumers) AND ("Abstract":application OR "Abstract":applications OR "Abstract":"App" OR "Abstract":"Apps" OR "Abstract": system OR "Abstract":service))&highlight=true&returnFacets=ALL&returnType=SEARCH&matchPubs=true&sortType=newest) | Abstract | ("Abstract":"AI" OR "Abstract":"artificial intelligence" OR "Abstract":"ML" OR "Abstract":"machine learning" OR "Abstract":"DL" OR "Abstract":"deep learning") AND ("Abstract":medical OR "Abstract":healthcare OR "Abstract":health) AND ("Abstract":consumer OR "Abstract":consumers) AND ("Abstract":application OR "Abstract":applications OR "Abstract":"App" OR "Abstract":"Apps" OR "Abstract": system OR "Abstract":service) | 113 |
| PubMed  [LINK](https://pubmed.ncbi.nlm.nih.gov/?term=(("AI"%5BTitle%2FAbstract%5D+OR+artificial+intelligence%5BTitle%2FAbstract%5D+OR+"ML"%5BTitle%2FAbstract%5D+OR+machine+learning%5BTitle%2FAbstract%5D+OR+"DL"%5BTitle%2FAbstract%5D+OR+deep+learning%5BTitle%2FAbstract%5D)+AND+(medical%5BTitle%2FAbstract%5D+OR+healthcare%5BTitle%2FAbstract%5D+OR+health%5BTitle%2FAbstract%5D)+AND+(consumer%5BTitle%2FAbstract%5D+OR+consumers%5BTitle%2FAbstract%5D)+AND+(application%5BTitle%2FAbstract%5D+OR+applications%5BTitle%2FAbstract%5D+OR+"App"%5BTitle%2FAbstract%5D+OR+"Apps"%5BTitle%2FAbstract%5D+OR+system%5BTitle%2FAbstract%5D+OR+systems%5BTitle%2FAbstract%5D+OR+service%5BTitle%2FAbstract%5D))&filter=simsearch3.fft&sort=date&size=200) | Title/Abstract | (("AI"[Title/Abstract] OR artificial intelligence[Title/Abstract] OR "ML"[Title/Abstract] OR machine learning[Title/Abstract] OR "DL"[Title/Abstract] OR deep learning[Title/Abstract]) AND (medical[Title/Abstract] OR healthcare[Title/Abstract] OR health[Title/Abstract]) AND (consumer[Title/Abstract] OR consumers[Title/Abstract]) AND (application[Title/Abstract] OR applications[Title/Abstract] OR "App"[Title/Abstract] OR "Apps"[Title/Abstract] OR system[Title/Abstract] OR systems[Title/Abstract] OR service[Title/Abstract])) | 354 |
| Google Scholar |  | (("AI" OR artificial intelligence OR "ML" OR machine learning OR "DL" OR deep learning) AND (medical OR healthcare OR health) AND (consumer OR consumers) AND (application OR applications OR "App" OR "Apps" OR system OR systems OR service)) | 100  (first 10 search-result pages) |
|  |  | **TOTAL**  **(After remove duplicates)** | 2,898 |
